# Crystal structure of ethyl 2-acetyl-3,7-dimethyl-5-(thio­phen-2-yl)-5*H*-thia­zolo[3,2-*a*]pyrimidine-6-carboxyl­ate

**DOI:** 10.1107/S2056989015010981

**Published:** 2015-06-13

**Authors:** N. L. Prasad, M. S. Krishnamurthy, Noor Shahina Begum

**Affiliations:** a Department of Studies in Chemistry, Central College Campus, Bangalore University, Bangalore 560 001, Karnataka, India

**Keywords:** crystal structure, fused pyrimidine derivative, hydrogen bonding, C—H⋯π inter­actions, π–π inter­actions

## Abstract

In the title compound, C_17_H_18_N_2_O_3_S_2_, the pyrimidine ring adopts a shallow sofa conformation, with the C atom bearing the axially-oriented thio­phene ring as the flap [deviation = 0.439 (3) Å]. The plane of the thio­phene ring lies almost normal to the pyrimidine ring, making a dihedral angle of 79.36 (19)°. In the crystal, pairs of very weak C—H⋯O hydrogen bonds link the mol­ecules related by twofold rotation axes, forming *R*_2_^2^(18) rings, which are in turn linked by another C—H⋯O inter­action, forming chains of rings along [010]. In addition, weak C—H⋯π(thio­phene) inter­actions link the chains into layers parallel to [001] and π–π inter­actions with a centroid–centroid distance of 3.772 (10) Å connect these layers into a three-dimensional network.

## Related literature   

For the biological activities of fused pyrimidine derivatives, see: Atwal *et al.* (1991 ▸); Kappe *et al.* (1997 ▸); Singh *et al.* (2011 ▸); Ozair *et al.* (2010 ▸); Hayam *et al.* (2010 ▸). For related structures, see: Prasad *et al.* (2014 ▸); Nagarajaiah *et al.* (2012 ▸).
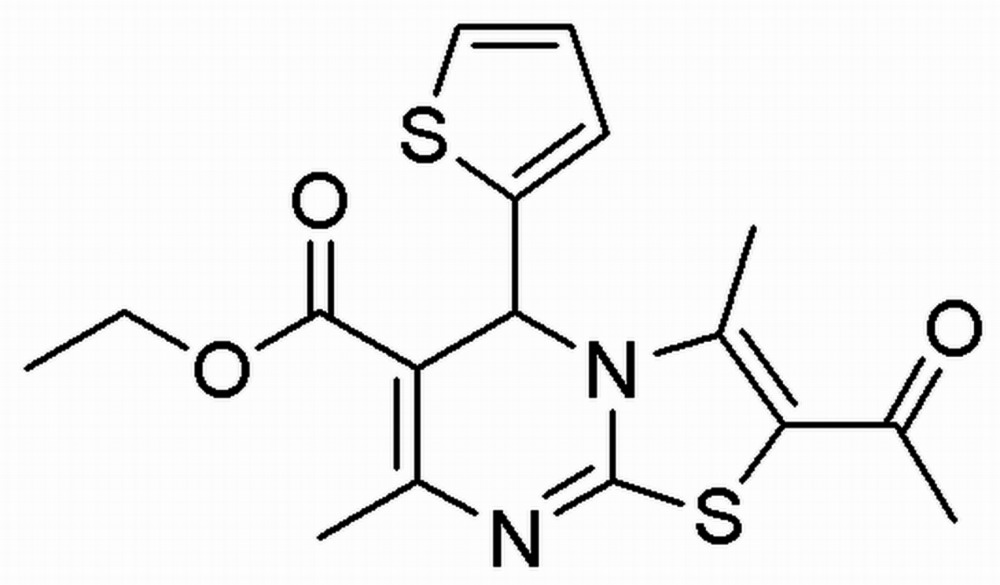


## Experimental   

### Crystal data  

C_17_H_18_N_2_O_3_S_2_
*M* *_r_* = 362.45
Monoclinic, 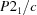

*a* = 7.8835 (10) Å
*b* = 14.4041 (19) Å
*c* = 15.231 (2) Å
β = 94.940 (4)°
*V* = 1723.2 (4) Å^3^
*Z* = 4
Mo *K*α radiation
μ = 0.33 mm^−1^
*T* = 100 K
0.18 × 0.16 × 0.16 mm

### Data collection  

Bruker SMART APEX CCD diffractometer
Absorption correction: multi-scan (*SADABS*; Bruker, 1998 ▸) *T*_min_ = 0.944, *T*_max_ = 0.950
12021 measured reflections
3038 independent reflections
1984 reflections with *I* > 2σ(*I*)
*R*_int_ = 0.046

### Refinement  

*R*[*F* ^2^ > 2σ(*F* ^2^)] = 0.065
*wR*(*F* ^2^) = 0.189
*S* = 1.00
3038 reflections
221 parameters
H-atom parameters constrained
Δρ_max_ = 0.51 e Å^−3^
Δρ_min_ = −0.28 e Å^−3^

### 

Data collection: *SMART* (Bruker,1998 ▸); cell refinement: *SAINT-Plus* (Bruker,1998 ▸); data reduction: *SAINT-Plus*; program(s) used to solve structure: *SHELXS97* (Sheldrick, 2008 ▸); program(s) used to refine structure: *SHELXL97* (Sheldrick, 2008 ▸); molecular graphics: *ORTEP-3 for Windows* (Farrugia, 2012 ▸) and *CAMERON* (Watkin *et al.*, 1996 ▸); software used to prepare material for publication: *WinGX* (Farrugia, 2012 ▸).

## Supplementary Material

Crystal structure: contains datablock(s) global, I. DOI: 10.1107/S2056989015010981/hb7433sup1.cif

Structure factors: contains datablock(s) I. DOI: 10.1107/S2056989015010981/hb7433Isup2.hkl

Supporting information file. DOI: 10.1107/S2056989015010981/hb7433Isup3.cml

**Fig. 1.**
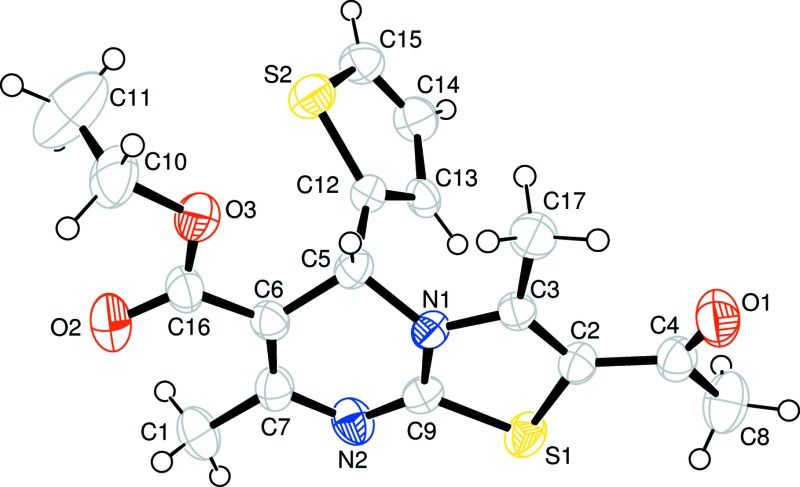
The molecular structure of the title compound with displacement ellipsoids drawn at the 50% probability level.

. DOI: 10.1107/S2056989015010981/hb7433fig1.tif
The mol­ecular structure of the title compound with displacement ellipsoids drawn at the 50% probability level.

**Fig. 2.**
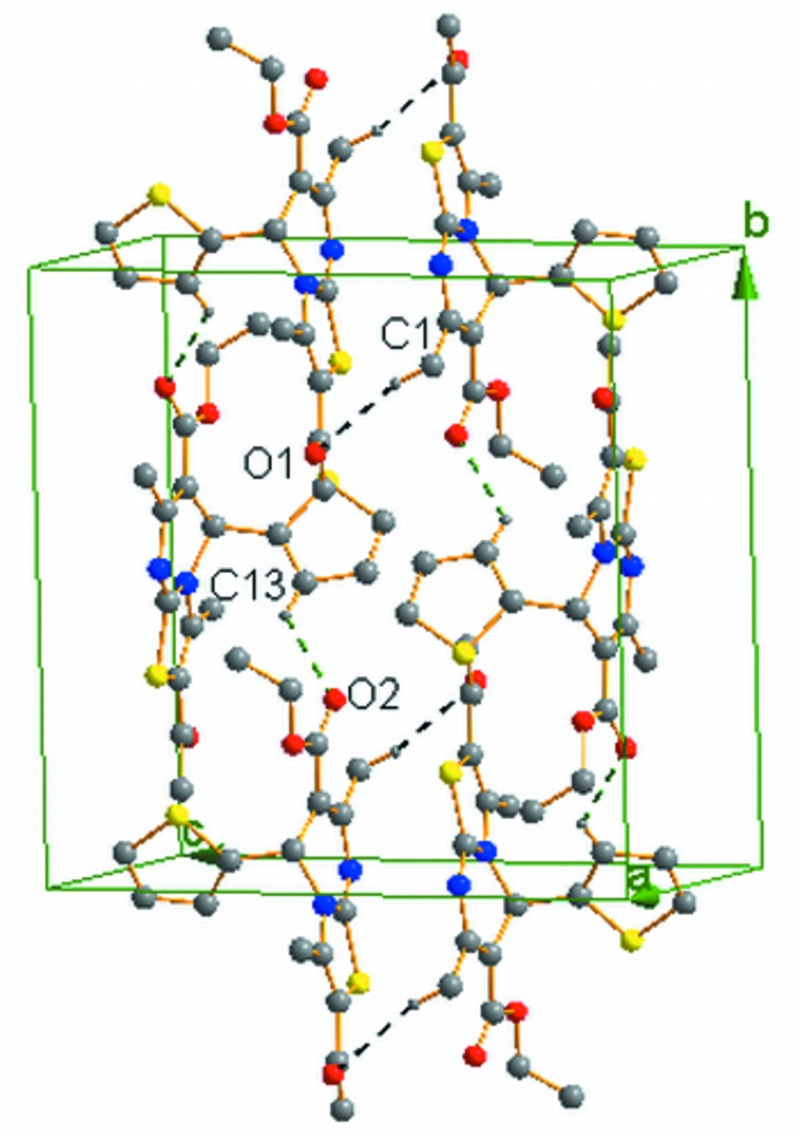
Unit-cell packing of the title compound showing C—H···O interactions as dotted lines. H atoms not involved in hydrogen bonding have been excluded.

. DOI: 10.1107/S2056989015010981/hb7433fig2.tif
Unit-cell packing of the title compound showing C—H⋯O inter­actions as dotted lines. H atoms not involved in hydrogen bonding have been excluded.

**Fig. 3.**
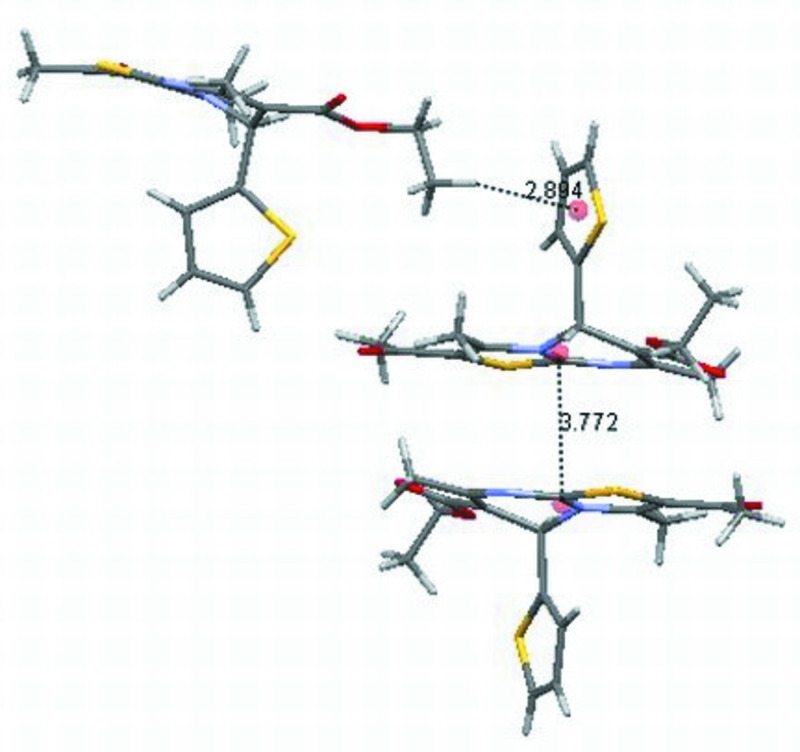
Unit-cell packing depicting the C—H···π and π–π interactions with dotted lines.

. DOI: 10.1107/S2056989015010981/hb7433fig3.tif
Unit-cell packing depicting the C—H⋯π and π–π inter­actions with dotted lines.

CCDC reference: 1405373

Additional supporting information:  crystallographic information; 3D view; checkCIF report

## Figures and Tables

**Table 1:** Hydrogen-bond geometry (, ) *Cg*1 is the centroid of the S2/C12C15 ring.

*D*H*A*	*D*H	H*A*	*D* *A*	*D*H*A*
C1H1*C*O1^i^	0.98	2.64	3.598(6)	166
C13H13O2^ii^	0.95	2.63	3.269(8)	125
C11H11*A* *Cg*1^iii^	0.98	2.89	3.693(2)	139

Symmetry codes: (i) 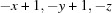; (ii) 
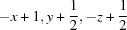
; (iii) 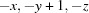.

## supplementary crystallographic information

### S1. Comment

Pyrimidine derivatives are important class of compounds which display number of pharmacological properties including antiviral, antitumour, antibacterial and antihypertensive effects (Atwal *et al.*, 1991; Kappe *et al.*, 1997). Pyrimidine has been subjected to a large variety of structural modifications in order to synthesize derivatives (Singh *et al.*, 2011) with different biological properties, among which, thiazole ring fused to pyrimidine ring resulting in thiazolopyrimidine is found to be more active (Ozair *et al.*, 2010; Hayam *et al.*, 2010). Herein, we report the crystal structure of the title compound (1). The bond lengths and angles in the title compound are in good agreement with the corresponding bond distances and angles reported in closely related structures (Nagarajaiah *et al.*, 2012; Prasad *et al.*, 2014). The molecular structure of the compound C_17_H_18_N_2_O_3_S_2_ is shown in Fig. 1. The thiophenyl ring at chiral carbon C5 is positioned axially and exactly bisects the pyrimidine ring with a dihedral angle of 82.973 (1)°. The thiazine and pyrimidine ring form a dihedral angle of 5.975 (1)°. In the central pyrimidine ring, the chiral carbon atom C6 is displaced by 0.3130 (4) Å and adopts a flattened *sofa* conformation. The exocyclic ester group at C6 adopts *cis* orientation with respect to C6=C7 double bond and the carboxyl group (C11/C10/O3/C16) is slightly deviating from the thiozolopyrimidine plane at -87.946 (6)°. The crystal structure is mainly stabilized by a variety of intermolecular C—H···O interactions. C1—H1C···O1 hydrogen bonds link the molecules related by twofold rotation axes, forming *R*^2^_2_(18) loops, which are in turn linked by C13—H13···O2 interactions to form chains of rings along [010] (Table.1; Fig. 2). In addition, weak C—H···π (thiophene) interactions of the type C11—H11A···*Cg* link the chains into layers parallel to [001] and π–π interactions between inversion-related thiazolopyrimidine rings with a centroid—centroid distance of 3.772 (10) Å connect these layers into a three-dimensional network (Fig. 3).

### S2. Experimental

A mixture of 6-methyl-4-thiophen-2-yl-2-thioxo-1,2,3,4-tetrahydro-pyrimidine-5- carboxylic acid ethyl ester (10 mmol) and 3-chloro-2,4-pentanedione (10 mmol) was refluxed in dry ethanol (20 mmol) for 12 h. The excess of solvent was distilled off and the solid hydrochloride salt that separated was collected by filtration, suspended in water and neutralized by aqueous sodium carbonate solution to yield the free base. The solution was filtered, the solid washed with water, dried and recrystallized from ethyl acetate to give the title compound (74% yield, mp 385 K). The compound was recrystallized by slow evaporation from 1:1 mixture of ethyl acetate and methanol, yielding pale- yellow blocks of the title compound.

### S3. Refinement

The H atoms were placed at calculated positions in the riding-model approximation with C—H = 0.95 Å, 1.00 Å and 0.96 Å for aromatic, methyne and methyl H-atoms respectively, with *U*_iso_(H) = 1.5*U*_eq_(C) for methyl H atoms and *U*_iso_(H) = 1.2*U*_eq_(C) for other hydrogen atoms.

### Crystal data

**Table:**

C_17_H_18_N_2_O_3_S_2_	*F*(000) = 760
*M**_r_* = 362.45	*D*_x_ = 1.397 Mg m^−^^3^
Monoclinic, *P*2_1_/*c*	Mo *K*α radiation, λ = 0.71073 Å
Hall symbol: -P 2ybc	Cell parameters from 3038 reflections
*a* = 7.8835 (10) Å	θ = 2.6–25.0°
*b* = 14.4041 (19) Å	µ = 0.33 mm^−^^1^
*c* = 15.231 (2) Å	*T* = 100 K
β = 94.940 (4)°	Block, yellow
*V* = 1723.2 (4) Å^3^	0.18 × 0.16 × 0.16 mm
*Z* = 4	

### Data collection

**Table:**

Bruker SMART APEX CCD diffractometer	3038 independent reflections
Radiation source: fine-focus sealed tube	1984 reflections with *I* > 2σ(*I*)
Graphite monochromator	*R*_int_ = 0.046
ω scans	θ_max_ = 25.0°, θ_min_ = 2.6°
Absorption correction: multi-scan (*SADABS*; Bruker, 1998)	*h* = −9→9
*T*_min_ = 0.944, *T*_max_ = 0.950	*k* = −17→17
12021 measured reflections	*l* = −17→18

### Refinement

**Table:**

Refinement on *F*^2^	Primary atom site location: structure-invariant direct methods
Least-squares matrix: full	Secondary atom site location: difference Fourier map
*R*[*F*^2^ > 2σ(*F*^2^)] = 0.065	Hydrogen site location: inferred from neighbouring sites
*wR*(*F*^2^) = 0.189	H-atom parameters constrained
*S* = 1.00	*w* = 1/[σ^2^(*F*_o_^2^) + (0.1059*P*)^2^ + 1.4984*P*] where *P* = (*F*_o_^2^ + 2*F*_c_^2^)/3
3038 reflections	(Δ/σ)_max_ < 0.001
221 parameters	Δρ_max_ = 0.51 e Å^−^^3^
0 restraints	Δρ_min_ = −0.28 e Å^−^^3^

### Special details

**Table:**

Geometry. All s.u.'s (except the s.u. in the dihedral angle between two l.s. planes) are estimated using the full covariance matrix. The cell s.u.'s are taken into account individually in the estimation of s.u.'s in distances, angles and torsion angles; correlations between s.u.'s in cell parameters are only used when they are defined by crystal symmetry. An approximate (isotropic) treatment of cell s.u.'s is used for estimating s.u.'s involving l.s. planes.
Refinement. Refinement of *F*^2^ against ALL reflections. The weighted *R*-factor *wR* and goodness of fit *S* are based on *F*^2^, conventional *R*-factors *R* are based on *F*, with *F* set to zero for negative *F*^2^. The threshold expression of *F*^2^ > 2σ(*F*^2^) is used only for calculating *R*-factors(gt) *etc*. and is not relevant to the choice of reflections for refinement. *R*-factors based on *F*^2^ are statistically about twice as large as those based on *F*, and *R*- factors based on ALL data will be even larger.

### Fractional atomic coordinates and isotropic or equivalent isotropic displacement parameters (Å^2^)

**Table:**

	*x*	*y*	*z*	*U*_iso_*/*U*_eq_	
S1	0.41155 (14)	0.67664 (8)	0.10428 (8)	0.0478 (4)	
S2	0.86975 (15)	0.38590 (8)	0.30180 (7)	0.0514 (4)	
N1	0.6122 (4)	0.5389 (2)	0.11268 (19)	0.0343 (8)	
C6	0.5337 (5)	0.3792 (3)	0.1355 (2)	0.0384 (10)	
O1	0.8379 (4)	0.7993 (2)	0.0552 (2)	0.0610 (9)	
C9	0.4445 (5)	0.5583 (3)	0.1190 (2)	0.0378 (10)	
N2	0.3249 (4)	0.5002 (3)	0.1340 (2)	0.0472 (9)	
C3	0.7148 (5)	0.6131 (3)	0.0965 (2)	0.0345 (9)	
C13	0.6889 (5)	0.5373 (3)	0.2994 (2)	0.0378 (10)	
H13	0.6144	0.5869	0.2815	0.045*	
O2	0.4904 (5)	0.2161 (2)	0.1141 (3)	0.0753 (11)	
C2	0.6282 (5)	0.6938 (3)	0.0902 (3)	0.0386 (10)	
C16	0.5843 (6)	0.2820 (3)	0.1255 (3)	0.0482 (11)	
C17	0.9012 (5)	0.5964 (3)	0.0884 (3)	0.0521 (12)	
H17A	0.9151	0.5420	0.0511	1.000*	
H17B	0.9592	0.5855	0.1470	1.000*	
H17C	0.9508	0.6509	0.0618	1.000*	
C12	0.7368 (5)	0.4638 (3)	0.2456 (2)	0.0359 (9)	
C7	0.3696 (6)	0.4072 (3)	0.1363 (3)	0.0434 (10)	
C5	0.6754 (5)	0.4494 (3)	0.1488 (2)	0.0359 (9)	
H5	0.7734	0.4286	0.1162	0.043*	
C10	0.8235 (8)	0.1808 (3)	0.1127 (4)	0.0683 (15)	
H10A	0.7431	0.1448	0.0724	0.082*	
H10B	0.9319	0.1866	0.0849	0.082*	
C14	0.7725 (6)	0.5240 (3)	0.3865 (3)	0.0499 (11)	
H14	0.7602	0.5662	0.4335	0.060*	
C4	0.6955 (6)	0.7881 (3)	0.0792 (3)	0.0447 (11)	
C1	0.2188 (6)	0.3437 (3)	0.1387 (3)	0.0592 (13)	
H1A	0.1198	0.3796	0.1538	1.000*	
H1B	0.2444	0.2954	0.1832	1.000*	
H1C	0.1940	0.3149	0.0808	1.000*	
C8	0.5863 (7)	0.8697 (3)	0.0997 (4)	0.0686 (15)	
H8A	0.5407	0.8599	0.1569	1.000*	
H8B	0.4920	0.8758	0.0538	1.000*	
H8C	0.6552	0.9264	0.1019	1.000*	
C15	0.8682 (6)	0.4481 (3)	0.3959 (3)	0.0484 (11)	
H15	0.9293	0.4306	0.4499	0.058*	
O3	0.7530 (4)	0.2735 (2)	0.1262 (2)	0.0560 (8)	
C11	0.8530 (10)	0.1323 (5)	0.1948 (4)	0.110 (3)	
H11A	0.8940	0.0694	0.1839	0.166*	
H11B	0.7466	0.1286	0.2235	0.166*	
H11C	0.9387	0.1656	0.2332	0.166*	

### Atomic displacement parameters (Å^2^)

**Table:**

	*U*^11^	*U*^22^	*U*^33^	*U*^12^	*U*^13^	*U*^23^
S1	0.0422 (7)	0.0400 (7)	0.0619 (7)	0.0079 (5)	0.0093 (5)	0.0074 (5)
S2	0.0591 (8)	0.0471 (7)	0.0467 (7)	0.0086 (5)	−0.0026 (5)	0.0011 (5)
N1	0.0349 (19)	0.0318 (18)	0.0364 (18)	0.0033 (15)	0.0036 (14)	0.0039 (14)
C6	0.043 (2)	0.038 (2)	0.033 (2)	−0.0054 (19)	−0.0006 (17)	−0.0010 (17)
O1	0.061 (2)	0.047 (2)	0.075 (2)	−0.0107 (16)	0.0111 (18)	0.0040 (16)
C9	0.037 (2)	0.039 (2)	0.037 (2)	0.0067 (19)	0.0031 (17)	0.0017 (18)
N2	0.038 (2)	0.048 (2)	0.057 (2)	−0.0058 (18)	0.0122 (17)	−0.0046 (18)
C3	0.038 (2)	0.036 (2)	0.029 (2)	−0.0036 (19)	0.0006 (16)	0.0010 (16)
C13	0.048 (2)	0.035 (2)	0.030 (2)	−0.0105 (18)	−0.0020 (18)	−0.0038 (17)
O2	0.077 (3)	0.041 (2)	0.106 (3)	−0.0134 (19)	−0.006 (2)	−0.0007 (19)
C2	0.041 (2)	0.036 (2)	0.039 (2)	−0.0001 (18)	0.0054 (18)	0.0045 (18)
C16	0.062 (3)	0.036 (2)	0.045 (3)	−0.009 (2)	−0.005 (2)	−0.0013 (19)
C17	0.036 (2)	0.051 (3)	0.070 (3)	0.001 (2)	0.008 (2)	0.008 (2)
C12	0.036 (2)	0.031 (2)	0.040 (2)	0.0005 (17)	0.0011 (17)	0.0016 (17)
C7	0.051 (3)	0.039 (3)	0.041 (2)	−0.006 (2)	0.0069 (19)	0.0014 (18)
C5	0.042 (2)	0.032 (2)	0.034 (2)	0.0055 (18)	0.0034 (17)	−0.0009 (16)
C10	0.085 (4)	0.042 (3)	0.078 (4)	0.013 (3)	0.008 (3)	−0.014 (3)
C14	0.057 (3)	0.051 (3)	0.042 (2)	0.000 (2)	0.004 (2)	−0.009 (2)
C4	0.055 (3)	0.040 (2)	0.039 (2)	−0.004 (2)	0.003 (2)	0.0020 (19)
C1	0.051 (3)	0.057 (3)	0.070 (3)	−0.022 (2)	0.013 (2)	0.001 (2)
C8	0.087 (4)	0.038 (3)	0.083 (4)	0.008 (3)	0.019 (3)	−0.005 (2)
C15	0.052 (3)	0.054 (3)	0.038 (2)	−0.001 (2)	−0.0011 (19)	0.003 (2)
O3	0.060 (2)	0.0390 (18)	0.067 (2)	0.0087 (15)	−0.0048 (16)	−0.0080 (15)
C11	0.157 (7)	0.091 (5)	0.086 (5)	0.063 (5)	0.027 (4)	0.017 (4)

### Geometric parameters (Å, º)

**Table:**

S1—C9	1.736 (4)	C17—H17B	0.9902
S1—C2	1.757 (4)	C17—H17C	0.9902
S2—C15	1.692 (4)	C12—C5	1.525 (5)
S2—C12	1.714 (4)	C7—C1	1.502 (6)
N1—C9	1.363 (5)	C5—H5	1.0000
N1—C3	1.375 (5)	C10—C11	1.433 (7)
N1—C5	1.471 (5)	C10—O3	1.467 (5)
C6—C7	1.355 (6)	C10—H10A	0.9900
C6—C16	1.468 (6)	C10—H10B	0.9900
C6—C5	1.508 (6)	C14—C15	1.328 (6)
O1—C4	1.221 (5)	C14—H14	0.9500
C9—N2	1.296 (5)	C4—C8	1.506 (6)
N2—C7	1.385 (5)	C1—H1A	0.9828
C3—C2	1.348 (5)	C1—H1B	0.9828
C3—C17	1.505 (6)	C1—H1C	0.9828
C13—C12	1.410 (5)	C8—H8A	0.9913
C13—C14	1.442 (6)	C8—H8B	0.9913
C13—H13	0.9500	C8—H8C	0.9913
O2—C16	1.207 (5)	C15—H15	0.9500
C2—C4	1.472 (6)	C11—H11A	0.9800
C16—O3	1.335 (5)	C11—H11B	0.9800
C17—H17A	0.9902	C11—H11C	0.9800
			
C9—S1—C2	91.04 (19)	C6—C5—C12	113.0 (3)
C15—S2—C12	91.6 (2)	N1—C5—H5	109.1
C9—N1—C3	116.3 (3)	C6—C5—H5	109.1
C9—N1—C5	116.9 (3)	C12—C5—H5	109.1
C3—N1—C5	124.2 (3)	C11—C10—O3	110.9 (4)
C7—C6—C16	123.5 (4)	C11—C10—H10A	109.5
C7—C6—C5	119.8 (4)	O3—C10—H10A	109.5
C16—C6—C5	116.7 (4)	C11—C10—H10B	109.4
N2—C9—N1	127.2 (4)	O3—C10—H10B	109.5
N2—C9—S1	123.7 (3)	H10A—C10—H10B	108.0
N1—C9—S1	109.1 (3)	C15—C14—C13	114.8 (4)
C9—N2—C7	116.2 (4)	C15—C14—H14	122.6
C2—C3—N1	112.4 (3)	C13—C14—H14	122.6
C2—C3—C17	128.6 (4)	O1—C4—C2	120.4 (4)
N1—C3—C17	119.0 (3)	O1—C4—C8	121.0 (4)
C12—C13—C14	108.1 (4)	C2—C4—C8	118.6 (4)
C12—C13—H13	126.0	C7—C1—H1A	109.8
C14—C13—H13	125.9	C7—C1—H1B	109.7
C3—C2—C4	128.2 (4)	H1A—C1—H1B	109.2
C3—C2—S1	111.2 (3)	C7—C1—H1C	109.8
C4—C2—S1	120.5 (3)	H1A—C1—H1C	109.2
O2—C16—O3	121.8 (4)	H1B—C1—H1C	109.2
O2—C16—C6	126.6 (5)	C4—C8—H8A	110.6
O3—C16—C6	111.5 (4)	C4—C8—H8B	110.6
C3—C17—H17A	110.5	H8A—C8—H8B	108.3
C3—C17—H17B	110.5	C4—C8—H8C	110.6
H17A—C17—H17B	108.4	H8A—C8—H8C	108.3
C3—C17—H17C	110.5	H8B—C8—H8C	108.3
H17A—C17—H17C	108.4	C14—C15—S2	112.9 (3)
H17B—C17—H17C	108.4	C14—C15—H15	123.5
C13—C12—C5	125.8 (3)	S2—C15—H15	123.5
C13—C12—S2	112.6 (3)	C16—O3—C10	118.1 (4)
C5—C12—S2	121.6 (3)	C10—C11—H11A	109.5
C6—C7—N2	121.9 (4)	C10—C11—H11B	109.5
C6—C7—C1	125.3 (4)	H11A—C11—H11B	109.5
N2—C7—C1	112.8 (4)	C10—C11—H11C	109.5
N1—C5—C6	108.3 (3)	H11A—C11—H11C	109.5
N1—C5—C12	108.2 (3)	H11B—C11—H11C	109.5
			
C3—N1—C9—N2	−179.9 (4)	C5—C6—C7—N2	11.1 (6)
C5—N1—C9—N2	−17.4 (6)	C16—C6—C7—C1	8.2 (6)
C3—N1—C9—S1	−0.2 (4)	C5—C6—C7—C1	−169.4 (4)
C5—N1—C9—S1	162.2 (2)	C9—N2—C7—C6	9.1 (6)
C2—S1—C9—N2	179.7 (4)	C9—N2—C7—C1	−170.4 (4)
C2—S1—C9—N1	0.0 (3)	C9—N1—C5—C6	33.4 (4)
N1—C9—N2—C7	−6.0 (6)	C3—N1—C5—C6	−165.7 (3)
S1—C9—N2—C7	174.4 (3)	C9—N1—C5—C12	−89.4 (4)
C9—N1—C3—C2	0.4 (5)	C3—N1—C5—C12	71.5 (4)
C5—N1—C3—C2	−160.6 (3)	C7—C6—C5—N1	−31.2 (5)
C9—N1—C3—C17	−179.8 (3)	C16—C6—C5—N1	151.0 (3)
C5—N1—C3—C17	19.2 (5)	C7—C6—C5—C12	88.6 (4)
N1—C3—C2—C4	175.6 (4)	C16—C6—C5—C12	−89.2 (4)
C17—C3—C2—C4	−4.2 (7)	C13—C12—C5—N1	20.6 (5)
N1—C3—C2—S1	−0.4 (4)	S2—C12—C5—N1	−162.0 (3)
C17—C3—C2—S1	179.8 (3)	C13—C12—C5—C6	−99.3 (4)
C9—S1—C2—C3	0.2 (3)	S2—C12—C5—C6	78.1 (4)
C9—S1—C2—C4	−176.1 (3)	C12—C13—C14—C15	−1.3 (5)
C7—C6—C16—O2	2.5 (7)	C3—C2—C4—O1	16.3 (7)
C5—C6—C16—O2	−179.8 (4)	S1—C2—C4—O1	−168.1 (3)
C7—C6—C16—O3	179.4 (4)	C3—C2—C4—C8	−162.5 (4)
C5—C6—C16—O3	−2.8 (5)	S1—C2—C4—C8	13.1 (5)
C14—C13—C12—C5	178.8 (4)	C13—C14—C15—S2	0.7 (5)
C14—C13—C12—S2	1.3 (4)	C12—S2—C15—C14	0.1 (4)
C15—S2—C12—C13	−0.8 (3)	O2—C16—O3—C10	−0.2 (6)
C15—S2—C12—C5	−178.5 (3)	C6—C16—O3—C10	−177.3 (3)
C16—C6—C7—N2	−171.2 (4)	C11—C10—O3—C16	−87.9 (6)

### Hydrogen-bond geometry (Å, º)

Cg1 is the centroid of the S2/C12–C15 ring.

**Table:**

*D*—H···*A*	*D*—H	H···*A*	*D*···*A*	*D*—H···*A*
C1—H1*C*···O1^i^	0.98	2.64	3.598 (6)	166
C13—H13···O2^ii^	0.95	2.63	3.269 (8)	125
C11—H11*A*···*Cg*1^iii^	0.98	2.89	3.693 (2)	139

Symmetry codes: (i) −*x*+1, −*y*+1, −*z*; (ii) −*x*+1, *y*+1/2, −*z*+1/2; (iii) −*x*, −*y*+1, −*z*.

## References

[bb1] Atwal, K. S., Swanson, B. N., Unger, S. E., Floyd, D. M., Moreland, S., Hedberg, A. & O’Reilly, B. C. (1991). *J. Med. Chem.* **34**, 806–811. 10.1021/jm00106a048 1995904

[bb3] Bruker. (1998). *SMART*, *SAINT-Plus* and *SADABS.* Bruker AXS Inc., Madison, Wisconsin, USA.

[bb4] Farrugia, L. J. (2012). *J. Appl. Cryst.* **45**, 849–854.

[bb5] Hayam, H. S., Eman, M. H. M. & Eman, R. K. (2010). *Synth. Commun.* **40**, 2712–2722.

[bb7] Kappe, C. O., Fabian, W. M. F. & Semones, M. A. (1997). *Tetrahedron*, **53**, 2803–2816.

[bb6] Nagarajaiah, H., Khazi, I. M. & Begum, N. S. (2012). *J. Chem. Sci.* **124**, 847–855.

[bb8] Ozair, A., Suroor, A. K., Nadeem, S. & Waquar, A. (2010). *Med. Chem. Res.* **19**, 1245–1258.

[bb9] Prasad, N. L., Krishnamurthy, M. S., Nagarajaiah, H. & Begum, N. S. (2014). *Acta Cryst.* E**70**, o1204. 10.1107/S1600536814023162 PMC4257338 25484830

[bb10] Sheldrick, G. M. (2008). *Acta Cryst.* A**64**, 112–122. 10.1107/S0108767307043930 18156677

[bb11] Singh, S., Schober, A., Gebinoga, M. & Alexander Gross, G. (2011). *Tetrahedron Lett.* **52**, 3814–3817.

[bb12] Watkin, D. J., Prout, C. K. & Pearce, L. J. (1996). *CAMERON.* Chemical Crystallography Laboratory, University of Oxford, England.

