# Supplementary figures and images for: Crystal structure of ethyl 2-acetyl-3,7-dimethyl-5-(thio­phen-2-yl)-5H-thia­zolo[3,2-a]pyrimidine-6-carboxyl­ate
Source: Acta Crystallogr E Crystallogr Commun. 2015 Jun 13;71(Pt 7):o477–8. doi: 10.1107/S2056989015010981 (PMC4518970; doi:10.1107/S2056989015010981)

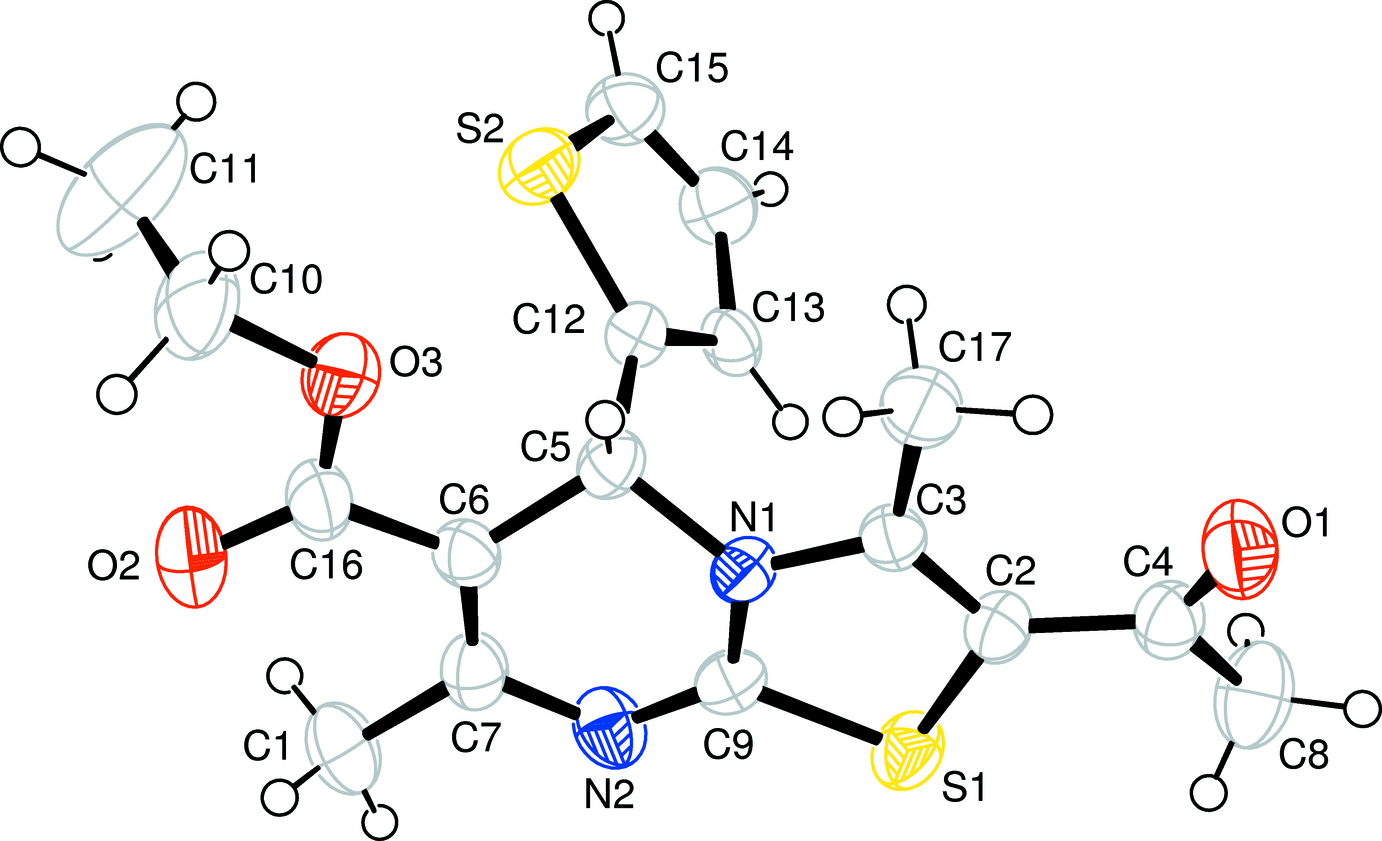

Supplement: Supplementary file 4 [file e-71-0o477-fig1.tif]

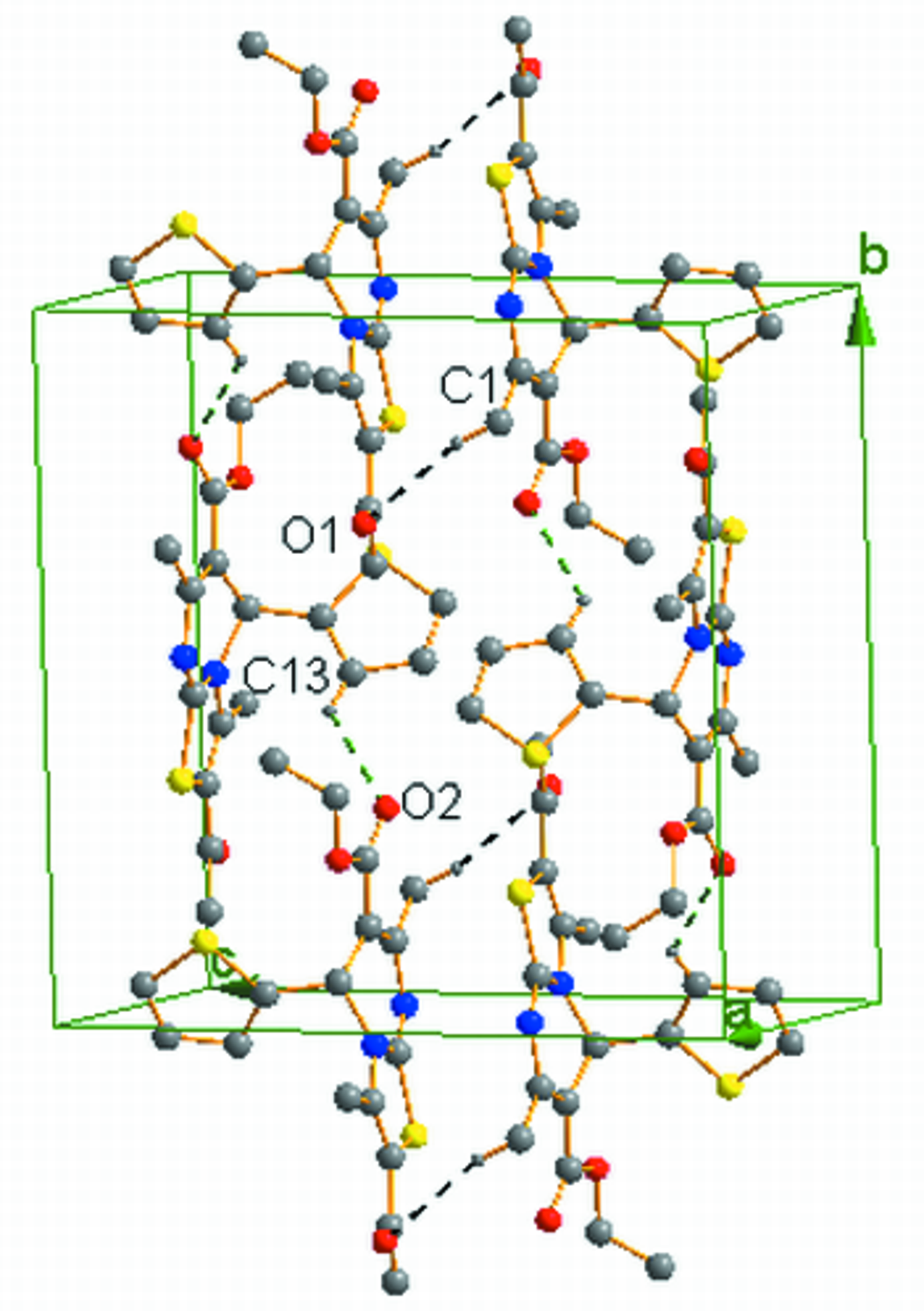

Supplement: Supplementary file 5 [file e-71-0o477-fig2.tif]

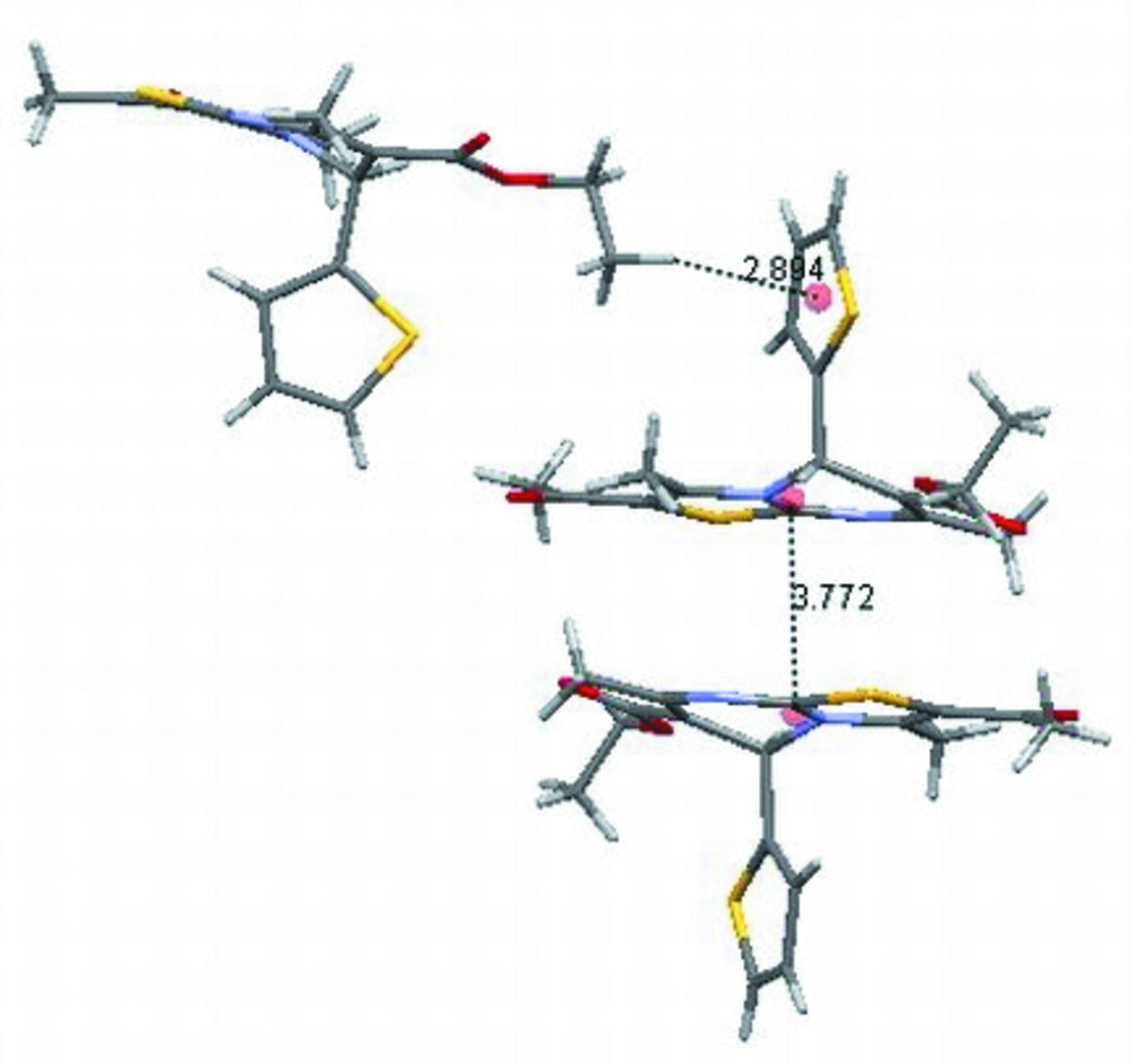

Supplement: Supplementary file 6 [file e-71-0o477-fig3.tif]
